# Role of the Treatment of Post-Concussion Syndrome in Preventing Long-Term Sequela Like Depression: A Systematic Review of the Randomized Controlled Trials

**DOI:** 10.7759/cureus.18212

**Published:** 2021-09-23

**Authors:** Tamil Poonkuil Mozhi Dhandapani, Ishan Garg, Anjli Tara, Jaimin N Patel, Jerry Lorren Dominic, Jimin Yeon, Marrium S Memon, Sanjay Rao Gergal Gopalkrishna Rao, Seif Bugazia, Safeera Khan

**Affiliations:** 1 Medicine, California Institute of Behavioral Neurosciences & Psychology, Fairfield, USA; 2 General Surgery, California Institute of Behavioral Neurosciences & Psychology, Fairfield, USA; 3 Internal Medicine, California Institute of Behavioral Neurosciences & Psychology, Fairfield, USA; 4 Research, California Institute of Behavioral Neurosciences & Psychology, Fairfield, USA

**Keywords:** post-concussion syndrome, post-concussive symptoms, chronic post-concussion syndrome, mild traumatic brain injury, depression

## Abstract

Traumatic brain injury of any severity can result in post-concussion syndrome (PCS). Although the post-concussive symptoms are complex, there is an emerging scientific consensus regarding the initiation of the treatment for these symptoms to improve quality of life and prevent long-term effects. The objective of this systematic review is to assess the comprehensive interventions used for the PCS and it aims to appraise if these interventions could prevent the development of depression as a complication. This research has used randomized controlled trials (RCTs) that evaluate the treatment of PCS and its effect on long-term complications like depression. We searched PubMed/MEDLINE, PubMed Central, Cochrane Central Register of Controlled Trials (CENTRAL), and EMBASE from January 1, 2016 to May 31, 2021 for our literature search. A quality check was conducted on the identified studies using the Cochrane risk of bias quality assessment tool (modified Cochrane RoB 2). In total, we included 11 RCTs and used Preferred Reporting Items for Systematic Reviews and Meta-Analyses (PRISMA) 2020 guidelines for the reporting of this systematic review. Most of the studies reinforced early initiation of the treatment by providing education to the patients and conducting their risk assessment. Strong evidence for the multidisciplinary treatment consisting of cognitive-behavioral therapy, psychoeducation, and physiotherapy is emphasized by some studies. More studies with a longer follow-up period are required to assess the effectiveness of intervention more accurately on depression. Regardless, this study will discuss guidelines and provide direction to physicians. It will help in developing future guidelines by addressing the clinical gaps in the implementation of these guidelines.

## Introduction and background

Mild traumatic brain injury (mTBI) is increasingly becoming a common health problem, affecting 600-1,200 per 100,000 people each year [1]. It may be caused by a motor vehicle collision, falls, assaults, strikes by/against an object, and sports and recreational injuries [2]. Although the majority of patients recover in a few weeks or months, a minority (15-25%) develop post-concussion syndrome (PCS) [3]. According to the International Classification of Diseases, Tenth Revision (ICD10), PCS is defined as a syndrome that follows a head injury severe enough to cause loss of consciousness and includes three or more out of eight symptoms such as headache, dizziness, fatigue, irritability, insomnia, impaired concentration, memory difficulties, and intolerance of stress, emotion, or alcohol [4].

Given the misconception that concussion does not need treatment from healthcare professionals, coupled with the perceived stigma of going through mental health care, the mTBI sustained individuals will be susceptible to develop maladaptive coping strategies. The symptoms may go on to become more prolonged [5]. Prolonged post-concussion syndrome (PPCS) is associated with poor outcomes resulting in lower community reintegration, higher healthcare utilization, higher economic cost, greater cognitive burden, and elevated reporting of psychiatric symptoms [6]. Particularly, depressive disorders are very common and seem to be important as their prevalence continues to be high in chronic stages of traumatic brain injury (TBI) [7,8]. A study by Potter et al. has reinforced the importance of preventing depression as the aforementioned poor outcomes are maintained via vicious cycles by the depression [9]. The ongoing debate on PCS is probably due to inconsistent terminology, various definitions [10], different diagnostic criteria [5], heterogeneity in disease outcome, as well as a variety of interventions [10,11]. Nevertheless, healthcare professionals acknowledge the significance of timely assessment, diagnosis, and treatment [12]. Considering this fact, various guidelines for the treatment of PCS are being evolved. But the area of implementation of these guidelines in clinical practice and their effect on the long-term complications are less explored.

The purpose of this review is to evaluate interventions used to treat PCS and find evidence to support that this intervention could prevent the development of depression. We reviewed the effectiveness of the various treatment modalities currently available for the PCS. We tried to understand the effect of these interventions on the development of depression and other neurobehavioral sequelae. The review will help evaluate the inconsistent clinical practice and provides evidence-based guidelines for implementation in clinical practice.

## Review

Methods

This systematic review was carried out by adhering to the Preferred Reporting Items for Systematic Reviews and Meta-Analyses (PRISMA) 2020 checklist [13].

Search Strategy

Four databases, PubMed/MEDLINE, PubMed Central, Cochrane Central Register of Controlled Trials (CENTRAL), and EMBASE, were searched for relevant articles. The final Medical Subject Heading (MeSH) search strategy used for PubMed is post-concussion syndrome OR post-concussive symptoms OR chronic post-concussion syndrome OR mild traumatic brain injury OR ("Post-Concussion Syndrome/classification"[Majr] OR "Post-Concussion Syndrome/complications"[Majr] OR "Post-Concussion Syndrome/diagnosis"[Majr] OR "Post-Concussion Syndrome/history"[Majr] OR "Post-Concussion Syndrome/prevention and control"[Majr] OR "Post-Concussion Syndrome/rehabilitation"[Majr] OR "Post-Concussion Syndrome/therapy"[Majr]) AND depression OR sadness OR melancholy OR ("Depression/diagnosis"[Majr] OR "Depression/etiology"[Majr] OR "Depression/physiopathology"[Majr]). We used the specific keywords (post-concussion syndrome and depression) in combination to search for the relevant papers in the Cochrane Library and EMBASE.

Screening of Articles

Titles and abstracts were screened independently by the two authors (TD and IG). The third author (AT) was involved in a discussion to resolve the conflicts, concerns, and doubts. The full text of relevant articles was retrieved and checked for eligibility criteria.

Inclusion and Exclusion Criteria

We only chose the randomized control trials (RCTs) published from January 1, 2016 to May 31, 2021. In addition, only studies published in the English language were included. Among the studies chosen, we ensured that all patients included were experiencing any of the symptoms of PCS or were at risk of developing PCS following TBI and were at least 18 years of age. We also included papers relevant to the research question regarding the long-term sequela of PCS, depression. Articles related to animal studies and the pediatric population were excluded.

Assessment of Study Quality

An assessment of the risk of bias was done on the selected studies using the Cochrane risk of bias tool (modified Cochrane RoB 2) and categorized as a high, low, or unclear risk of bias in each of the seven domains.

Data Extraction

Data extraction for the 11 included studies was done by the two authors (TD and IG). We extracted the objective of the study, participant characteristics (sample size, age group, severity of TBI, and time since TBI), characteristics of intervention and control group, follow-up period, outcome assessed in the study, and the result. The data considered to best reflect the relevance of the study were extracted and discussed by the two authors.

Results

**Figure 1:**
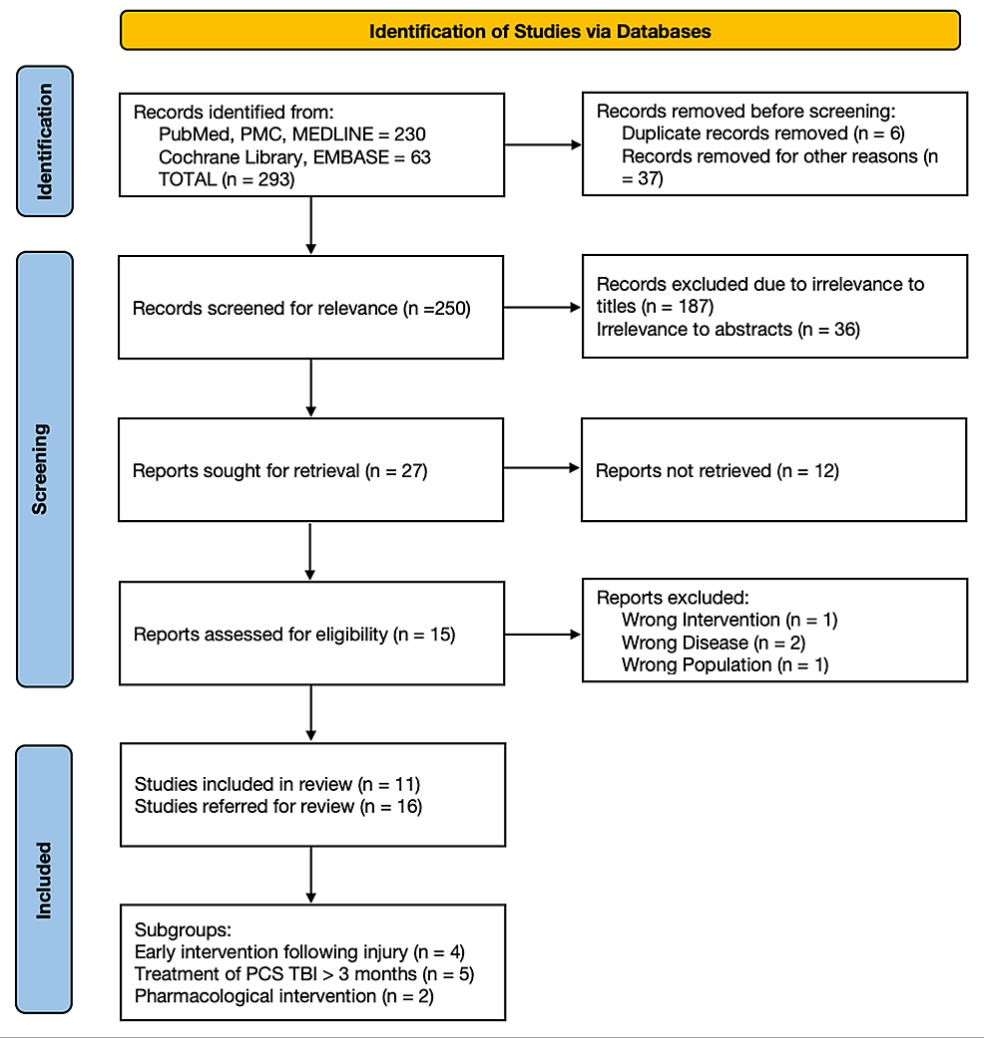
PRISMA 2020 flow diagram. PMC = PubMed Central; PCS = post-concussion syndrome; TBI = traumatic brain injury.

A total of 32,720 articles were identified using the search strategy mentioned in the method section. It was shortlisted to 293 RCTs after applying five filters (RCTs, publication date from January 1, 2016 to May 31, 2021, human species, English language, and 18 plus age group), from which duplicates were removed manually. The articles were then screened based on the title and abstract related to the research question. Papers were also filtered based on the eligibility criteria and availability of full text. A total of 11 articles were identified to be eligible for review and quality assessment was done for these articles. A PRISMA flow diagram is given in Figure 1. Table 1 shows the outcome of the Cochrane risk of bias tool and Table 2 exhibits the characteristics of the included RCTs.

**Table 1:** Summary of the Cochrane risk of bias tool. L = low risk of bias; U = unclear risk of bias; H = high risk of bias.

Author	Random sequence generation	Allocation concealment	Selective reporting	Other sources of bias	Blinding of participants and personnel	Blinding of outcome assessment	Incomplete outcome data
Silverberg et al. [1]	L	L	U	L	L	L	L
Bosch et al. [3]	L	L	U	L	U	L	L
Rytter et al. [5]	L	L	U	L	H	L	L
Jurick et al. [6]	L	U	U	L	U	U	L
Jorge et al. [8]	L	U	U	L	L	U	L
Potter et al. [9]	L	U	U	L	H	U	L
Scheenen et al. [14]	L	L	U	L	U	U	L
Mortimer et al. [15]	L	L	U	L	U	L	L
Bell et al. [16]	L	L	U	L	U	L	L
Vas et al. [17]	L	L	U	L	L	L	L
Theadom et al. [18]	L	L	U	L	L	L	H

**Table 2:** Characteristics of included studies. TBI = traumatic brain injury; mTBI = mild traumatic brain injury; RPQ = Rivermead Post-Concussion Symptoms Questionnaire; PHQ-9 = Patient Health Questionnaire - 9 Item; PVT = Performance Validity Test; CPT = cognitive processing therapy; PTSD = post-traumatic stress disorder; CogSMART = Cognitive Symptom Management and Rehabilitation Therapy; BDI-II = Beck Depression Inventory-II; PTA = post-traumatic amnesia; HADS = Hospital Anxiety and Depression Scale; S-REHAB = Specialized Interdisciplinary Rehabilitation; MDI = Major Depression Inventory; PPCS = prolonged PCS; MLC901 = NeuroAID, a herbal antioxidant; QOL = quality of life; PST = problem-solving treatment; EO = education only; TAU = treatment as usual; SM = service members; PSQI = Pittsburgh Sleep Quality Index; CBT = cognitive behavioral therapy; PCS = post-concussion syndrome; SMART = Strategic Memory Advanced Reasoning Training; MDD = major depressive disorder; MINI = Mini-International Neuropsychiatric Interview; BHW = Brain Health Workshop.

Author/year	Objectives	Participant characteristics	Time since TBI	Description of interventions	Outcome assessed	Results
Silverberg et al. (2020) [1]	1. Document undertreatment. 2. Delivering an implementation intervention to family physicians	n = 148; 18 to 60 years; mTBI; male = 52	<3 months post-injury	1. Experimental clinics-received guideline implementation tool (tailored follow-up letters). 2. Control clinics received generic follow-up letters	1. Patient recall of physician actions. 2. Blinded chart audits. 3. Patient-reported clinical outcomes. The outcome was measured using RPQ and PHQ-9	The experimental group reported fewer symptoms on RPQ compared with those in the control group
Jurick et al. (2019) [6]	1. Invalid versus valid range on PVTs show similar benefits from psychotherapy. 2. Psychotherapy improves PVT performance	n = 100; mean age 34.39 years; veterans with mild-moderate TBI, PTSD; male = 89	Mean 5.36 years	1. Standard CPT or CPT with embedded cognitive rehabilitation strategies from CogSMART (SMART-CPT)	Mental health symptoms using three checklists including BDI-II	The PVT-Pass group demonstrated greater symptom reduction than the PVT-Fail group
Bosch et al. (2019) [3]	To evaluate the effectiveness of implementation intervention compared with the dissemination of a guideline on the management of mTBI patients	n = 1,943; 18 years or older; mTBI; male = 1,022	Within 24 h of injury	Implementation intervention compared with the dissemination of a guideline	Appropriate screening for PTA using a tool. The outcome was measured using HADS and RPQ	PTA is more appropriately assessed in the intervention group
Mortimer et al. (2018) [15]	Economic evaluation from a health sector perspective for the above intervention	n = 1,943; 18 years or older; mTBI; male = 1,022	Within 24 h of injury	Implementation intervention compared with the dissemination of a guideline	PTA screening is the primary outcome for the economic evaluation. The outcome was measured using HADS and RPQ	Intervention is more costly and more effective
Rytter et al. (2018) [5]	S-REHAB would result in a greater reduction of PPCS and improve subjective symptoms	n = 89; 18-65 years; mild TBI; male = 30	>6 months with PCS	S-REHAB vs. standard care in PPCS patients	1. Assess post-concussion symptoms using RPQ. 2. Headache, fatigue, and depression. The outcome was measured using MDI	S-REHAB is more effective in reducing PPCS
Theadom et al. (2018) [18]	Safety and effects of MLC901	n = 78; 18-65 years; mild-moderate TBI; male = 39	1-12 months post-TBI	MLC901 vs. placebo	Cognitive functioning neurobehavioral sequelae, QOL, anxiety, and depression. The outcome was measured using RPQ and HADS	Improvement of cognitive function with six months of treatment
Bell et al. (2017) [16]	1. PST in addition to treatment as usual would result in greater improvement in PCS compared to EO plus TAU. 2. Effect of PST on daily function, QOL, depression, and insomnia	n = 356; mean age 29.35 years; SM with mTBI; male = 332	Trauma within previous 24 months	PST vs. EO (problem-solving treatment by telehealth and education only)	1. Psychological distress and PCS using RPQ. 2. Sleep, depression, PTSD, and physical functioning. The outcome was measured using PSQI, PHQ-9, and others	Telehealth-PST appears well accepted and reduces psychological distress and could be a useful adjunct treatment
Scheenen et al. (2017) [14]	1. To examine the effectiveness of CBT intervention compared to telephonic counseling (TC). 2. To improve functional outcomes and lower post-traumatic complaints	n = 91; 18-65 years; mTBI; male = 46	Starting four to six weeks after trauma	CBT intervention compared to TC	1. Return to work (RTW) six and 12 months after trauma. 2. Depression and anxiety. The outcome was measured using HADS	1. No difference in RTW between the two groups. 2. Fewer symptoms and full recovery in TC group at 12 months
Potter et al. (2016) [9]	Impact of CBT to reduce PCS and to increase QOL and health status	n = 46; 18-65 years; mild-moderate-severe TBI; male = 25	>6 months post TBI	CBT vs. control waiting list group	1. QOL and post-concussion symptoms were assessed. 2. Anxiety and fatigue were assessed. The secondary outcome was measured using HADS	Improvement in QOL in patients with prolonged symptoms
Vas et al. (2016) [17]	1. To examine the benefits of SMART. 2. Effect on psychological health	n = 60; 19-65 years; mTBI; male = 32	>6 months post TBI	SMART strategies vs. BHW	Gist reasoning, executive functions, memory, and psychological health. The outcome was measured using BDI-II	Gain in gist reasoning in the SMART group compared to BHW
Jorge et al. (2016) [8]	Assess the efficacy of sertraline in preventing MDD following TBI	n = 94; 18-85 years; any TBI; male = 56	Completed PTA recovery within four weeks post TBI	Sertraline 100 mg/d for 24 weeks vs. placebo	Time to onset of an MDD and diagnosed using MINI	Sertraline appears to be efficacious to prevent the onset of MDD

The outcome was evaluated in the 11 RCTs with 3,005 patients. The duration of the intervention ranges from five weeks to 24 weeks in the studied RCTs as follows: Scheenen et al. (five weeks), Vas et al. (eight weeks), Potter et al. and Jurick et al. (12 weeks), Rytter et al. (22 weeks), and Bell et al. (24 weeks). The pharmacological intervention in the trial by Theadom et al. and Jorge et al. was conducted for 24 weeks.

Discussion

Post-concussion syndrome is a chronic health condition and is considered problematic [9]. Despite this, the treatment guidelines are not well known and are widely disseminated [3]. This systematic review provides comprehensive evidence of treatment efficacy in PCS, and we aim to help policymakers and researchers address this evidence gap in the future. Consistent with the previous evidence, the prevention of post-concussion symptoms following any traumatic brain injury is an important priority [14].

Prevention of PCS With Early Interventions

Effective management starts at the emergency department (ED) where the challenge of safe discharge of the TBI-sustained patient is encountered. Bosch et al. conducted a study of 31 EDs to evaluate a theory-informed and targeted implementation intervention called Neurotrauma Evidence Translation (NET) intervention. The study reinforced the effectiveness of multi-faceted, targeted, theory-informed interventions compared to single-component, non-targeted other interventions in increasing the uptake of recommendations. Three clinical practice recommendations deemed important by NET guidelines are (a) assessing post-traumatic amnesia (PTA) in ED using a validated tool; (b) ascertaining the proper use and timing of computed tomography (CT) imaging; and (c) providing verbal patient information as well as patient education handouts consisting of advice and reassurance during discharge from ED [3]. Another study by Mortimer et al., which was conducted alongside the NET trial, evaluated the cost-effectiveness of this targeted theory-based, multi-faceted intervention. It pointed out that even though the management of mTBI in the ED is more efficient with NET intervention, it is unlikely to be cost-effective [15]. Nonetheless, ED places a crucial role in initiating the treatment for mTBI patients, and further research may be needed to study cost-effective evaluation strategies.

Follow-up with a family physician and other primary care practitioners should be arranged as they play a unique role in implementing and promoting early treatment to prevent chronicity in mTBI. A practice guideline was developed for primary care practitioners by incorporating the best available evidence by an interdisciplinary team supported by Ontario Neurotrauma Foundation (ONF). An RCT studying 114 different primary care clinics measured the improved outcome in mTBI patients following the family physician's behavior change. The study included a guideline implementation tool that was sent to the family physician. It had a tailored letter with ONF guidelines specific to the primary symptoms as suggested by the participant's positive screening test. The study documented fewer post-concussion symptoms in the experimental clinics but the treatment of insomnia was higher in the control clinics [1]. Scheenen et al. mentioned in their study that patients at risk of persistent complaints should be targeted for preventive cognitive behavioral therapy (CBT). They used Head Injury Symptom Checklist (HISC), which is similar to the Rivermead Post-Concussion Symptoms Questionnaire (RPQ), to measure the post-traumatic complaints, and it included further questions on sleeping problems and anxiety. The study emphasized that providing education with CBT in the early phase of the injury is more effective [14]. All these studies support the fact that early intervention should include establishing the diagnosis, initial assessment, and treatment targeting symptoms, as well as providing verbal and written education about anticipated recovery. A summary of the studies on early intervention is shown in Table 3.

**Table 3:** Studies on early intervention. PTA = post-traumatic amnesia; CT = computed tomography; ED = emergency department; NET = Neurotrauma Evidence Translation; RPQ = Rivermead Post-Concussion symptoms Questionnaire; CBT = cognitive behavioral therapy; TC = telephonic counseling; RTW = return to work.

Author/year	Intervention	Follow-up period	Outcome
Bosch et al. (2019) [3]	To increase the uptake of three key recommendations (PTA tool, CT scan rule, information booklet) in ED. The intervention group received all the below while the control group received only the first two steps. 1. A copy of guidelines is provided. 2. Data collection reminder is given. 3. A one-hour meeting discussed key recommendations, intervention components, and anticipated barriers. 4. An opinion leader team identified. 5. A one-day training related to the key recommendation and their role for nurses and opinion leaders. 6. Opinion leaders give training to staff members over three months. 7. Provision of materials and tools.	1. NET sample: only clinical practice outcome at two months. 2. NET plus sample: both practice outcome and patient outcome at four to ten months	1. More appropriately assessed for PTA at two months (clinical practice outcome). 2. Small effect on anxiety level (patient outcome)
Mortimer et al. (2018) [15]	Economic evaluation from a health sector perspective for the above intervention	1. NET sample: two months post-intervention 2. NET plus sample: one-month post-discharge	NET intervention is more costly and more effective
Silverberg et al. (2020) [1]	1. Experimental clinics-received guideline implementation tool (tailored follow-up letters). 2. Control clinics received generic follow-up letters	The outcome assessed after one month and three months	The experimental group reported fewer symptoms on the RPQ compared with those in the control group
Scheenen et al. (2017) [14]	CBT intervention (CBTi) five hourly sessions focusing on (i) psychoeducation on mTBI, (ii) replace dysfunctional with realistic belief, and (iii) providing coping strategies. Compared to telephonic counseling (TC) five sessions providing information and reassurance.	Return to work (RTW) six and 12 months after trauma	1. No difference in RTW between the two groups. 2. Fewer symptoms and full recovery in TC group at 12 months

Treatment of Post-Concussion Symptoms and its Role in Preventing Depression

The prolonged duration of symptoms greater than three months following TBI usually warrants treatment that incorporates interdisciplinary interventions. Various trials have shown improvements in the prolonged symptoms using cognitive rehabilitation, cognitive behavioral therapy (CBT), psychoeducation, and physiotherapy.

CBT: The CBT component in the multidisciplinary intervention plays an essential role in the treatment of PCS. The CBT alone could have an impact without other interventions. As shown in the research done by Potter et al., there is an improvement in the quality of life with a program of only 12 hours of therapist counseling, especially in those completing treatment more quickly [9]. Despite the evidence of treatment effect on anxiety and fatigue, there was no evidence noted for effect on depression [9]. As demonstrated by this trial, CBT has a vital role in the treatment of PCS. Further, Bell et al. have supported the effectiveness of problem-solving treatment in service members, which allowed participants to select the problems to work on from the list generated during the initial telehealth sessions. Hence, the entire 12 telehealth sessions were the participant-centered model. The study showed at six months of follow-up that the PST group reported greater improvements in psychological distress and also noted short-term improvement in depression [16]. As we see, telehealth is emerging as a widely accepted model of practice for common diseases, and this particular scenario for the treatment of PCS via telehealth should be further researched and explored. CBT focuses on identifying key symptoms and prioritizing them during treatment. Hence, CBT can positively impact the quality of life of patients with PCS.

Integrative neurocognitive therapies: Cognitive impairment is a common disability of the PCS. Integrative neurocognitive therapies are widely accepted strategies to address these impairments. It helps to improve the cognitive deficits along with guiding strategies to adapt to life post-injury [17]. Researchers constantly develop, test, and identify cognitive training protocols to improve the daily function of individuals with TBI. Examples of such programs include Goal-Oriented Attentional Self-Regulation (GOALS), problem-solving treatment (PST), goal management training (GMT), and Strategic Memory Advanced Reasoning Training (SMART) [17]. Vas et al. have shown the cognitive benefits of SMART in an mTBI population at a chronic stage. Notable among the secondary outcome is psychological health. Psychological health, measured as the self-report of depressive symptoms and stress-related behaviors, showed significant gains on the SMART group [17]. This points out that interventions could prevent psychiatric sequelae of PCS like depression with multidimensional and inter-related strategies. Another study by Jurick et al. has reinforced this evidence and has emphasized the relations between the inadequate engagement of testing in veterans with mTBI and the increased reporting of psychiatric symptoms. They compared two groups (a) cognitive processing therapy (CPT) combined with the components of cognitive training from Cognitive Symptom Management and Rehabilitation Therapy (CogSMART) to (b) the standard CPT [6]. Consistent with their previous RCTs, the first group SMART-CPT demonstrated greater improvements in mental health symptoms (PCS) and quality of life, even though both groups showed improvements following treatment. In addition, the study included a performance validity test (PVT) to measure the engagement of veterans in the neuropsychological test evaluation. Although both PVT pass and PVT fail groups showed clinically significant improvement in mental health after the treatment, the PVT fail group demonstrated better test engagement at follow-up [6]. The study provides empirical support to enroll the PVT fail group in trauma-focused treatment as they may benefit from assessment after treatment [6].

Interdisciplinary rehabilitation: A treatment plan that incorporates multiple therapies and is tailored based on the patient's symptoms would be the best approach to follow in PCS management. The recent developments support the provision of active rehabilitation for the prolonged PCS (PPCS). The trial by Rytter et al. has shown the effectiveness of the specialized interdisciplinary rehabilitation (S-REHAB) compared to the standard care for PCS. This 22-week program focused on education and return to work. It included both individual and group sessions [5]. The trial successfully targets cognitive, emotional, and physical domains along with interpersonal skills. The study concluded improvements in social functioning, increased level of activity life satisfaction coupled with a decrease in mental fatigue, which is a valuable finding entailing a need for individually tailored rehabilitation for the sufferers of PCS [5]. A summary of the studies on the treatment of post-concussion syndrome is shown in Table 4.

**Table 4:** Studies on treatment of PCS. CBT = cognitive behavioral therapy; QOL = quality of life; PST = problem-solving treatment; EO = education only; SMART = Strategic Memory Advanced Reasoning Training; BHW = Brain Health Workshop; CPT = cognitive processing therapy; CogSMART = Cognitive Symptom Management and Rehabilitation Therapy; TBI = traumatic brain injury; PVT = performance validity test; S-REHAB = Specialized Interdisciplinary Rehabilitation; PPCS = prolonged post-concussion syndrome.

Author/year	Intervention	Follow-up period	Outcome
Potter et al. (2016) [9]	CBT: Twelve weekly one-hour sessions focusing on (i) identifying problems and psychoeducational and (ii) problem-solving and relapse prevention. This is compared to the control waiting list group.	At four months	Improvement in QOL in patients with prolonged symptoms.
Bell et al. (2017) [16]	PST by telehealth which includes 12 biweekly telephone calls for subject select problems and 12 educational brochures. This is compared with the EO group, which includes biweekly emails of brochures and 12 educational brochures.	At six months and 12 months	PST appears well accepted and reduces psychological distress. It could be a useful adjunct treatment.
Vas et al. (2016) [17]	SMART strategies focus on blocking distractions, engaging abstractions, and addressing problems (12 sessions of 18 hours in total over eight weeks). This is compared with BHW (12 sessions of 18 hours in total over eight weeks).	At three months	Gain in gist reasoning in the SMART group compared to BHW.
Jurick et al. (2019) [6]	CPT with embedded cognitive rehabilitation strategies from CogSMART (SMART-CPT) for 12 weeks including psychoeducation about TBI and simplified CPT worksheets. This is compared with standard CPT (12 weeks treatment).	At three months	The PVT-Pass group demonstrated greater symptom reduction than the PVT-Fail group.
Rytter et al. (2018) [5]	S-REHAB for 22 weeks which includes neuropsychological treatment, exercise therapy, and physiotherapeutic coaching. This is compared with standard care in PPCS patients (more variable depending on their local municipalities).	At six months	S-REHAB is more effective in reducing PPCS.

Pharmacological Interventions

Few studies are employing pharmacological intervention for the PCS in the search results. Also, we found pharmacological intervention targeted specific symptoms the patient identified [19]. Jorge et al. studied 96 patients to assess the efficacy of sertraline to prevent depression following TBI and found the number of need to treat to prevent depression is 5.9. Although the small sample size limits the study, it indicated antidepressants appear to be efficacious in delaying the onset of depression [8]. Theadom et al. also used a pilot RCT of 78 patients to evaluate the effects of NeuroAiD, an antioxidant, on cognitive functioning following TBI and reported evidence that NeuroAiD improved cognitive functioning [18].

Limitations

This review has a few limitations. First, this systematic review has included only RCTs. As PCS includes a number of symptoms, we could have missed reviewing few interventions used to treat PCS. Second, the condition is highly heterogeneous, and there are considerable variations in the selection of participants among the studies. However, most studies included mild to moderate TBI patients in their trials. Third, few studies had the risk of bias that could have pooled up.

Even so, bias in blinding participants in the rehabilitation studies, which studies the subjective symptoms, is an expected problem. Finally, the follow-up period for the studies is not too long to study the development of depression and other long-term sequelae. Taken together, future trials with a large population and long follow-up times will be required for supportive evidence.

## Conclusions

Annually, there is an increase in the incidence of PCS following TBI, and various causes that inflict the injury in combination with the rising awareness of PCS among the patients could play a role. This paper was conducted to find the clinical gaps in managing PCS and to determine factors in preventing long-term neurobehavioral problems. The review of the studies shows that management of PCS is associated with significant outcomes in improving the quality of life, decreasing the economic burden on the health system, and perhaps reducing the long-term complications, such as depression and anxiety. Treatment should be initiated as early as possible by providing education about the expected trajectory following TBI and identifying at-risk patients for long-term symptoms. As the PCS includes a group of symptoms, the primary symptom should be identified and given individualized treatment. In addition to these, the multidisciplinary team intervention would provide the best outcome. The interdisciplinary treatment, including but is by no means limited to cognitive behavioral therapy, psychoeducation, physiotherapy, should be incorporated in the treatment process. Future research should include a large population and a long follow-up period to synthesize evidence of long-term outcomes.
